# Antenna complexes protect Photosystem I from Photoinhibition

**DOI:** 10.1186/1471-2229-9-71

**Published:** 2009-06-09

**Authors:** Alessandro Alboresi, Matteo Ballottari, Rainer Hienerwadel, Giorgio M Giacometti, Tomas Morosinotto

**Affiliations:** 1 Laboratoire de Génétique et Biophysique des Plantes – UMR 6191 CEA-CNRS-Université de la Méditerranée, Marseille, France; 2 Dipartimento di Biotecnologie, Università di Verona, Verona, Italy; 3 Dipartimento di Biologia, Università di Padova, Padova, Italy

## Abstract

**Background:**

Photosystems are composed of two moieties, a reaction center and a peripheral antenna system. In photosynthetic eukaryotes the latter system is composed of proteins belonging to Lhc family. An increasing set of evidences demonstrated how these polypeptides play a relevant physiological function in both light harvesting and photoprotection. Despite the sequence similarity between antenna proteins associated with the two Photosystems, present knowledge on their physiological role is mostly limited to complexes associated to Photosystem II.

**Results:**

In this work we analyzed the physiological role of Photosystem I antenna system in *Arabidopsis thaliana *both *in vivo *and *in vitro*. Plants depleted in individual antenna polypeptides showed a reduced capacity for photoprotection and an increased production of reactive oxygen species upon high light exposure. *In vitro *experiments on isolated complexes confirmed that depletion of antenna proteins reduced the resistance of isolated Photosystem I particles to high light and that the antenna is effective in photoprotection only upon the interaction with the core complex.

**Conclusion:**

We show that antenna proteins play a dual role in *Arabidopsis thaliana *Photosystem I photoprotection: first, a Photosystem I with an intact antenna system is more resistant to high light because of a reduced production of reactive oxygen species and, second, antenna chlorophyll-proteins are the first target of high light damages. When photoprotection mechanisms become insufficient, the antenna chlorophyll proteins act as fuses: LHCI chlorophylls are degraded while the reaction center photochemical activity is maintained. Differences with respect to photoprotection strategy in Photosystem II, where the reaction center is the first target of photoinhibition, are discussed.

## Background

Photosynthesis is powered by light absorbed by chlorophyll (Chl) and carotenoid molecules bound to thylakoid membrane proteins. These pigment-binding proteins are organized in two supramolecular complexes: Photosystem (PS) I and II. Each Photosystem is composed of two different moieties: (i) the core complex, responsible for charge separation and the first steps of electron transport and (ii) the peripheral antenna system, which plays a role in light harvesting and transfer of excitation energy to the reaction center. Antenna polypeptides in green algae and plants are all members of a multigenic family of proteins called Lhc (Light harvesting complexes, [1,2]. PSI antenna system (LHCI) comprises four major proteins, Lhca1-4, while PSII antenna comprises six, Lhcb1-6 [1]. Additional Lhc sequences have been identified, namely Lhca5-6 and Lhcb7-8: the corresponding polypeptides, however, are less expressed and their physiological significance remains unclear [1,3].

Light is the energy source for photosynthesis but, if the rate of energy absorption exceeds the overall rate of electron transfer, it may cause photo-oxidation damages [4]. PSII is generally considered as the major target of this phenomenon (photoinhibition) as it is less stable than PSI under strong light treatments [5,6]. Several photoprotection mechanisms activated with different timescales, such as NPQ (Non Photochemical Quenching), xanthophyll cycle and acclimation were shown to be involved in PSII photoprotection. Interestingly, members of the multigenic family of antenna proteins have been shown to be involved to some extent in all these mechanisms [7-14]. A growing set of evidences, thus, supports the idea that antenna proteins, at least those associated to PSII, play a dual role and are involved both in light harvesting and photoprotection [9].

In contrast PSI is generally believed to be less sensitive to light stress [5] and its photoprotection mechanisms were less investigated. However, several recent evidences showed that PSI can also be targeted by photoinhibition, especially under chilling conditions and when the linear electron transport chain is unbalanced [15-20]. PSI photoprotection has been suggested to be mainly mediated by oxygen scavenging enzymes (e.g., superoxide dismutase and ascorbate peroxidase) which efficiently detoxify reactive species produced at the reducing side of Photosystem I [21]. A decreased ability of these enzymes to scavenge ROS at low temperatures was proposed to be the reason of the major PSI photo-sensitivity in chilling conditions [17]. In addition, it was shown that PSI reaction centre itself is very efficient in dissipating energy as heat [22-26].

Despite the sequence similarity with PSII antenna polypeptides, a possible role of PSI antenna polypeptides in photoprotection was never completely proven [27-30]. Recent evidences, however, suggested that PSI antenna might play a more relevant role than generally believed. In fact, Lhca depleted plants showed a large reduction in their fitness in a natural environment: all genotypes produced less seeds than WT under sunlight and the largest differences were observed in the case of ΔLhca2 and ΔLhca4 mutants [31]. Moreover, it was shown that carotenoids bound to Lhca polypeptides are very efficient in quenching Chl triplets, suggesting that the pigments bound to these complexes are well protected from radiation damages [32,33].

In this work we analyzed the role of LHCI polypeptides in photoprotection, using a combination of *in vivo *and *in vitro *approaches: we first compared the phenotype of plants depleted in individual Lhca polypeptides under strong illumination [34,35] and observed that reactive oxygen species (ROS) production was positively correlated with the reduction in PSI antenna size, suggesting a role of LHCI chlorophyll-proteins in photoprotection. This hypothesis was confirmed by experiments performed *in vitro *on isolated complexes, confirming that the *in vivo *phenotype was due to differences in the composition of the PSI-LHCI complexes. We also show that antenna complexes are the first target in PSI photoinhibition, suggesting they act as safety fuses in PSI photoprotection. When radiation damages cannot be avoided, their targeting to the LHCI antenna prevents damages at the reaction center, whose catalytic activity is thus stable even under high light conditions.

## Results

### Lhca depleted plants are defective in photoprotection

Despite their name, light harvesting complexes (Lhc) are not only involved in light capture but they also play a relevant role in photoprotection. Evidences supporting this idea, however, have been almost exclusively obtained for PSII antenna polypeptides, while the physiological role of Lhc complexes associated to PSI is still poorly understood. We analyzed plants depleted in Lhca proteins, whose absence drove to destabilization of the PSI antenna system at different extents, depending on the targeted gene product [34,35]. The largest effect was observed upon deletion of Lhca4, followed by Lhca2 and Lhca3. In the case of ΔLhca1 the effect was the weakest. However, this last statement must be considered as provisional since the only available mutant is a leaky allele, which still retains a significant levels of Lhca1 [34]. Functional and biochemical analyses showed that the PSI antenna size in these genotypes was in the following order: Δa4 < Δa2 = Δa3 < Δa1 [34,35]. In contrast to previous work, we used here a KO plant for Lhca2, instead of the previously characterized antisense plants [36]. However, as described in Additional file 1, biochemical characterization of PSI samples from these plants showed very similar results to data obtained on antisense Lhca2 plants.

**Figure 1:**
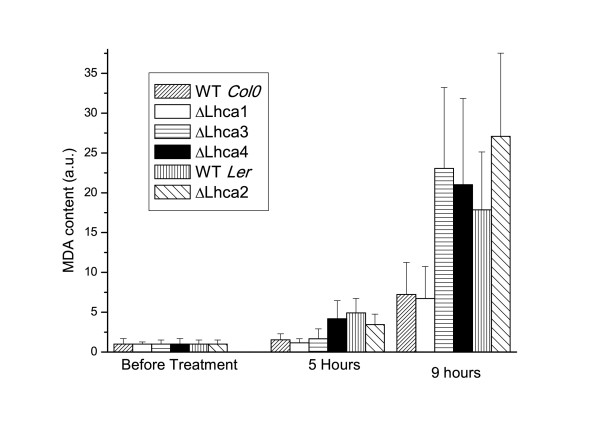
**Assessment of photoprotection ability of Lhca depleted plants**. Leaf disks cut from WT and ΔLhca1-4 plants were treated with high light at 4°C. MDA production, measuring the amount of reactive oxygen species produced during treatment, was evaluated before and after 5 and 9 hours of light treatment. SD is also reported, calculated from six to twelve replicates.

In order to evaluate the resistance to high light of these genotypes, leaf disks were cut from plants grown in control conditions and treated for 9 hours with strong light (900 μE) at 4°C. Under excess light conditions, reactive oxygen species (ROS) react with lipids producing lipid peroxides, which can be quantified by the amount of malondialdehyde (MDA) as measured by HPLC. MDA is a three-carbon, low molecular weight aldehyde produced by radical attack on polyunsaturated fatty acids: its quantification is an indication of the amount of lipid peroxidation and, indirectly, of the amount of ROS produced [37]. As shown in figure 1A, MDA quantification showed that three genotypes (ΔLhca2, 3 and 4) produce significantly larger amounts of lipid peroxides upon high light treatment with respect to their controls. Assessment of ROS production by MDA production thus suggests that the reduction of PSI antenna size brings about an increased sensitivity to high light. This effect is not due to a different Chl concentration in leaves since Chl concentration per fresh weight was found to be similar in WT and mutants (see Additional file 2). It is worth mentioning that we observed a different MDA production in Columbia and Landsberg erecta WT plants, consistently with previous observations that different *Arabidopsis *ecotypes have different resistance to stressful conditions [38,39].

Similar experiments were performed with whole plants instead of leaf disks: again, an increased bleaching and MDA production was observed in mutants, even if, due to the unavailability of a large 4°C growth chamber, high light treatments were performed at 22°C for a longer time (4 days) in order to obtain comparable MDA productions. Thereafter we focused our experiments on detached leaves for two main reasons: first, in the long term we could not avoid the activation of an acclimative response in plants and thus the activation of photoprotective mechanisms which might mask the effect of Lhca depletion. As an example, during acclimation the synthesis of several antioxidant molecules such as carotenoids, vitamin E, anthocyanins and other flavonoids might be differentially induced [40]. Second, since PSI is more sensitive to photoinhibition at chilling temperatures, low temperatures are more suited to analyze photoprotection mechanisms in this supercomplex [16,17].

### PSI particles with reduced antenna show increased photosensitivity

**Figure 2:**
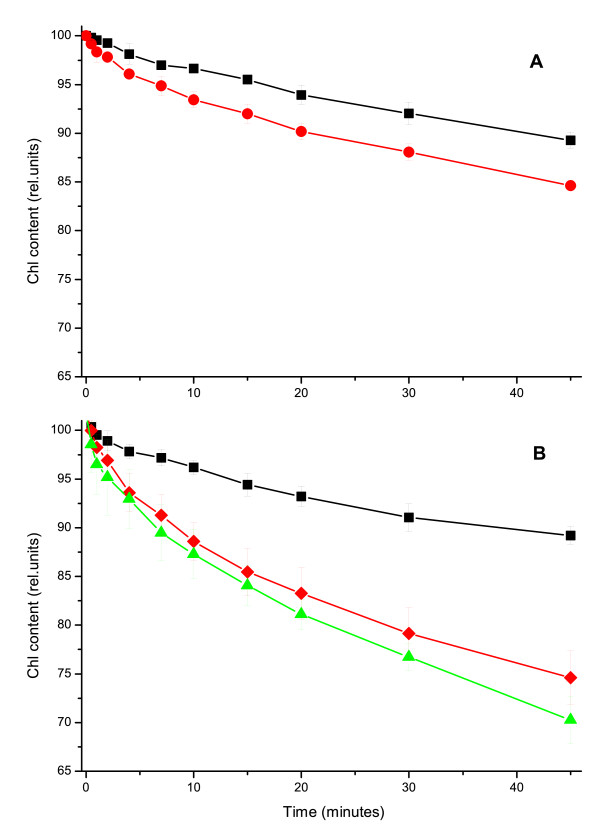
**Antenna association increases PSI particles resistance to high light treatment**. A) PSI particles purified from WT and Lhca4 depleted plants were illuminated with high light (1600 μE) at 10°C for 45 minutes. Absorption spectra were registered during the treatment and the absorption area is reported here (WT, black squares, ΔLhca4 red circles) after normalization of the value before the treatment to 100%. B) The same analysis was done comparing PSI-LHCI from WT (black squares) with isolated PSI core (red diamonds) and LHCI (green triangles). Samples concentrations were normalized to known PSI Chl to protein stoichiometries (170 Chls per PSI-LHCI, 102 per PSI core, 50 per LHCI). SD is also reported, calculated from at least three replicates.

In order to verify that the phenotype observed *in vivo *in different plants was indeed due to differences in the PSI-LHCI supercomplex composition and not to secondary effects of the mutation, we evaluated the resistance to high light of PSI-LHCI particles isolated from the different genotypes. These were purified from WT and mutants as in [35] and illuminated at 10°C with 1600 μE for 45 minutes, a treatment causing a significant Chl bleaching (around 10%) in PSI-LHCI complex. PSI particles isolated from ΔLhca4 plants, which are characterized by the smallest antenna size, when subjected to the same treatment, showed an increased Chl bleaching, as reported in figure 2A.

The observation that PSI with reduced antenna is less stable in response to high light treatment suggests that antenna proteins are indeed relevant in photoprotection of the complex. In order to confirm this result, we repeated the same photobleaching experiments by comparing PSI-LHCI particles with the PSI core and LHCI moieties purified from WT thylakoids. Results in figure 2B show that, consistent with the above results, PSI core is less resistant to illumination with respect to the whole PSI-LHCI supercomplex. This observation thus confirms the role of the antenna system in protecting PSI from excess illumination damages. However, data in figure 2B also show that this protecting effect is not due to an intrinsically higher resistance of LHCI: in fact, isolated LHCI is even more sensitive to high light than the PSI reaction center. To confirm this, we measured the bleaching kinetics of PSI core + LHCI mixed together, which showed an intermediate behavior with respect to isolated PSI core and LHCI (not shown).

These experiments were done using the same P700 concentrations, exploiting known PSI to Chl stoichiometries [41], in order to evidence differences between a PSI core with or without antenna complexes. We nevertheless verified that differences in concentration were not affecting the results: in experiments using equivalent Chl amounts isolated core and antenna moieties were more sensitive to high light treatment with respect to whole PSI-LHCI complexes (see Additional file 3).

### LHCI is effective in protecting Photosystem I catalytic activity

**Figure 3:**
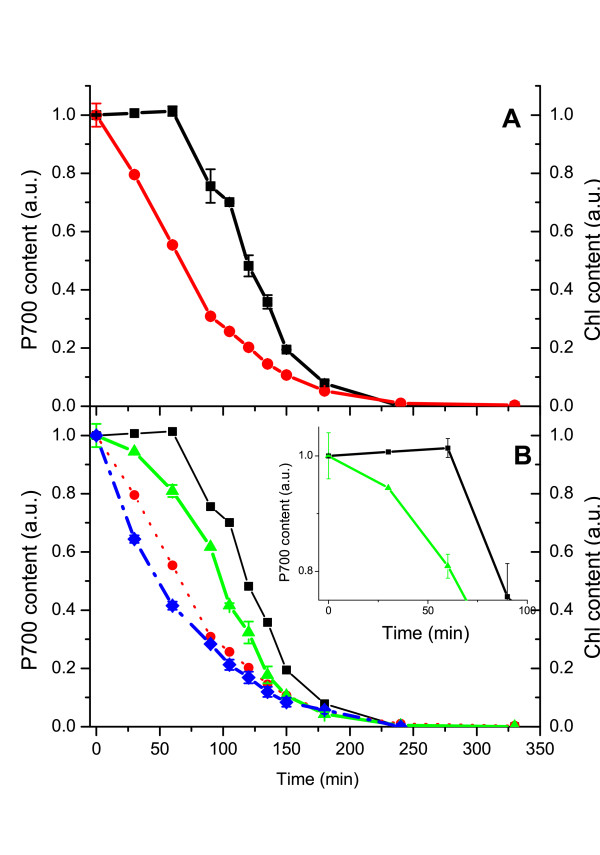
**LHCI association preserves PSI activity under high light conditions**. PSI-LHCI complex was treated with high light (1000 μE at 4°C). In (A) PSI-LHCI WT Chl bleaching (red circles) is reported together with the P700 content, determined from the P700^+ ^absorption at 435 nm (black squares). In (B) Chl bleaching (blue diamonds) and P700 content (green triangles) are reported for the experiments performed with PSI particles purified from ΔLhca4 plants. Data from WT are also reported for comparison (red and black for Chl bleaching and PSI activity respectively). Inset: comparison of decrease of P700 content in WT vs. ΔLhca4 plants. All data are the result of at least 3 experimental repetitions, for each P700 measurement three independent samples were pooled together. SD is also reported.

Data presented so far suggest that LHCI is effective in protecting PSI-LHCI from radiation damage. The observed protecting activity could be considered physiologically important only if it reduces damages to PSI core and preserves PSI capacity to perform charge separation. This is particularly relevant considering that PSI-LHCI was shown to miss an efficient mechanism for damage repair and PSI reaction center requires several days to recover from photoinhibition [42]. To test the antenna effect in light induced damages to reaction center during a high light treatment of isolated PSI-LHCI particles (1000 μE at 4°C for 270 minutes, see details in the material section) we estimated Chl amount and P700 concentration, determined by the P700^+ ^absorption at 435 nm (Figure 3). As expected, during the high light treatment a progressive Chl bleaching was observed. Very interestingly, however, the PSI capacity of charge separation showed a different behavior: in fact, initially it was not affected at all and started decreasing only after 60 minutes of treatment, when 30% of total PSI Chls were already bleached. This clearly proves that PSI-LHCI undergoes strong damages which do not affect its charge separation capacity: in this supercomplex, thus, other Chls are bleached before P700 is affected. It is worth mentioning that the P700^+ ^signal observed allows indirect detection of damages to other PSI core components such as Fe-S clusters: in fact, in case of damages to other cofactors involved in electron transport, P700^+ ^decay components decrease typically to 1 ms or less [43]. Time components of this magnitude were indeed observed during the experiment, but only in samples treated with more than 90 minutes of high light (not shown), suggesting the whole PSI core complex is stable in the first phase of light treatment.

In order to analyze the possibility that the antenna system actually plays a role in this process, the same analysis was performed on PSI particles purified from ΔLhca4 plants. In this case, we observed a faster Chl bleaching, as already pointed out, but also a faster decrease in concentration of photo-oxidizable P700 concentration, clearly showing that the reduction in antenna size reduces its protection from Chl bleaching but also from impairment of charge separation capacity. Moreover, in ΔLhca4 mutant photo-oxidizable P700 decreases together with Chl bleaching starting from the very beginning of the light treatment. These results clearly demonstrate that the antenna complex is needed in order to localize radiation damages far from the PSI reaction center. The relevance of the antenna is evident from data in the inset of figure 3B: while in WT photo-oxidizable P700 concentration is substantially unaffected after 60 minutes of light treatment, in ΔLhca4 plants in the same time period it is already reduced by 20%. Thus in most physiological conditions, the presence of the antenna prevents P700 from undergoing significant damages.

### LHCI is the first target of PSI photoinhibition

Results presented so far suggested that P700 Chls are not the first targets in PSI photoinhibition. In order to identify what these targets are, we analyzed in more detail the modification occurring in the PSI-LHCI super-complex during the strong illumination treatment.

**Figure 4:**
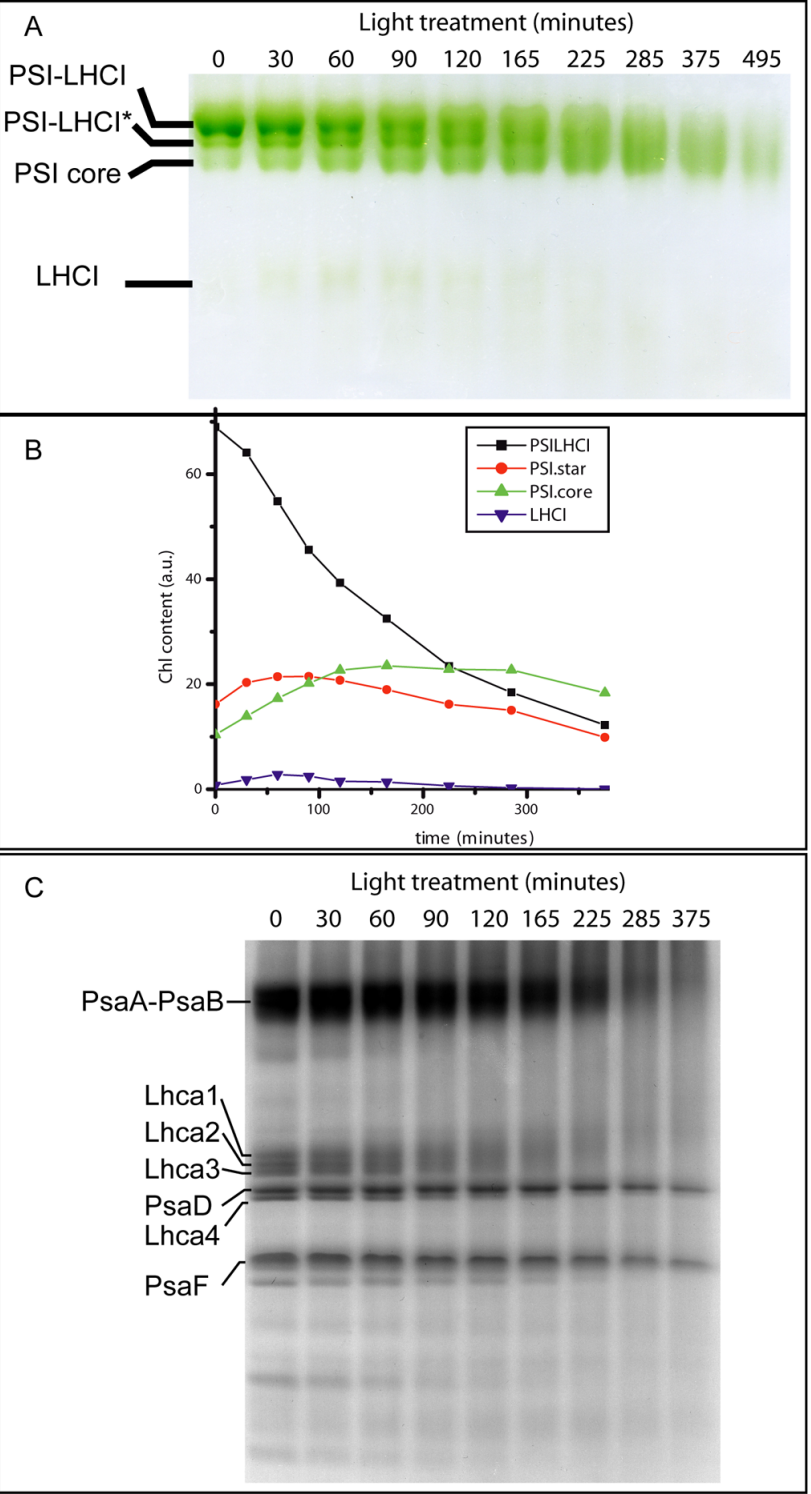
**LHCI is the first target in high light in PSI**. PSI was treated with 1000 μE at 4°C and biochemically characterized during the treatment. The composition in different pigment protein complexes was analyzed by non denaturing Deriphat PAGE (A). (B) Densitometric quantification of different bands, normalized to the total Chl content of the sample before treatment. C) Polypeptide composition of PSI particles during the illumination was analyzed by SDS PAGE. The bands corresponding to Lhca, PsaA-B, PsaD and PsaF polypeptides, as identified by western blotting analysis, are evidenced. Other bands corresponding to PSI core polypeptides are indicated.

The effect on PSI-LHCI supramolecular organization was first analyzed by non denaturing gel electrophoresis. In figure 4A the result of the separation is shown: three main green bands can be detected and, as indicated in the figure, they were identified as PSI-LHCI, PSI-LHCI* and PSI core by running a second dimension SDS PAGE (not shown). PSI-LHCI* represents a PSI population with reduced antenna, which was already observed in Lhca depleted plants [35]. In addition, a faint band corresponding to dimeric LHCI was also detectable in lanes 2–6. The presence of all three PSI core populations at time t0 can be explained by the detergent present in the running buffers, which causes some dissociation of the antenna from the core. Such a dissociation is not generally observed on thylakoids [35] and is probably due to the higher detergent/protein ratio when running isolated PSI particles. Despite this limitation, however, from figure 4A it clearly appears that, during the bleaching treatment, PSI-LHCI population is reduced progressively. On the contrary, the intensity of PSI core band increases in the first 100 minutes of illumination and then starts decreasing after 300 minutes. This clearly suggests that light treatment causes a dissociation of PSI-LHCI supercomplex. However, while consistent amounts of PSI core are detected, isolated LHCI is very low, suggesting that these pigment protein complexes are degraded first.

Densitometric quantification of the different bands, reported in figure 4B, confirms this view. PSI-LHCI constantly decreases during the light treatment. In a first phase this is correlated with the increase in PSI-LHCI* and PSI core populations, while free LHCI is hardly detectable. Thus, LHCI bound Chls are preferentially degraded in this phase, while PSI-LHCI* and PSI core are affected only later.

Light effects on polypeptides composition were also analyzed by loading on a SDS-PAGE samples at different stages of the light treatment. As shown in figure 4C, light induced protein degradation is observed as several bands become fainter in the SDS-PAGE pattern. Moreover, degradation products become visible as smeared bands. Lhca polypeptides are the most sensitive to this treatment: densitometric analysis showed a significant reduction (> 20%) of Lhca polypeptides after t3 (90 min). Instead, PSI core polypeptides like PsaB, which is known to be a main target of PSI core upon illumination [44], are more resistant to light treatment and show protein degradation only at t5 (165 min). Other PSI core polypeptides like PsaD and PsaF are even more stable and show relevant degradation only after t7 (285 min). By densitometric analysis we also compared the degradation rate of the different antenna polypeptides, but the differences were too small to be significant. This suggests that there are little differences in light susceptibility among the Lhca polypeptides and they are all affected simultaneously or in a very fast succession. Mutant phenotypes are thus rather due to differences in antenna size than due to the absence of a specific polypeptide.

## Discussion

### LHCI polypeptides protects PSI from excess light

In this work we analyzed the phenotype under high light of plants depleted in Lhca polypeptides. These mutants have a different LHCI content, ranging from around 5–10% of WT levels in ΔLhca4, 20–30% in ΔLhca2-3 to 50–60% in ΔLhca1 plants [34,35]. Plants with the strongest depletion in LHCI polypeptides showed higher lipid peroxide formation quantified as MDA derivates [37].

If we compare this measure with literature data on several mutants affecting photoprotection, although plant treatments are not equivalent, we observed that the phenotype of Lhca depleted plants is strong. In contrast, several mutants in the carotenoid biosynthesis or *PsbS *mutant, although affected in well known photoprotection mechanisms do not show significant differences in MDA production [45]. Consistent with this hypothesis of a relevant role of LHCI in photoprotection, plant fitness experiments in a natural environment showed that ΔLhca4 plants had the strongest phenotype with respect to all other Lhca or Lhcb depleted plants or even PsbS less plants [31].

The role of Lhca polypeptides in photoprotection has been confirmed by *in vitro *experiments. Both PSI particles isolated from ΔLhca4 depleted plants and purified PSI core showed a faster Chl bleaching under high light compared to PSI-LHCI WT particles. Isolated LHCI showed an even lower resistance to high light, implying that its photoprotective effect requires the interaction with PSI core: PSI-LHCI supercomplex, in fact, is more resistant to light treatment than both individual moieties.

In this respect it is worth reminding that PSI-LHCI supercomplex is peculiar because it contains a pigment complement, constituted by "Gap" and "Linker" Chls, which are bound at the interface between the core and the antenna moiety [41,46]. These Chls are specific of PSI and were shown to be implied in the linkage between core and antenna and in stabilizing the supercomplex structure [35]. Data presented here add new information to the picture showing that these pigments are also relevant for its photoprotection.

### LHCI acts as a safety valve for PSI activity

PSI with reduced antenna or isolated PSI core showed a lower resistance than PSI-LHCI, both *in vivo *and *in vitro*. The presence of the antenna system strongly influences the extent of radiation damage but also its localization. In fact, antenna complexes are the first target of radiation damages in PSI-LHCI: figure 4 is very clear in this respect, showing that during the first part of light treatment PSI core is stable while only the antenna moiety is affected. These results are also consistent with previous results showing the antenna sensitivity to high light in PSI, both *in vivo *[47] and *in vitro *[28,48].

This preferential degradation of LHCI upon illumination is effective in protecting PSI catalytic activity: during the first phase of light treatment, concentration of photo-oxidizable P700 is maintained despite a significant degradation of a fraction of Chl molecules. The LHCI role in PSI photoprotection is thus dual: its presence not only increases the overall resistance to high light, but also changes the localization of the radiation damage far from the reaction center, acting like a safety valve. In contrast to PSII, it appears that the subunits suffering radiation damages are antenna subunits while the reaction center is more stable.

It is interesting to underline that around 30% of Chls were already bleached before a significant decrease in P700 concentration was observed. Such a large Chl bleaching is very difficult to observe in leaves and, although this figure cannot be directly transferred *in vivo*, it suggests that in most physiological conditions PSI activity is efficiently protected.

Consistent with the hypothesis that radiation damage to the PSI core is a rare event, PSI repair mechanisms are known to be very slow: even after several days, recovery from PSI core photoinhibition is still incomplete [42,49,50]. Furthermore, recovery requires complete degradation of photoinhibited PSI and its complete re-synthesis [42], making the process energetically demanding. We may thus hypothesize that, in the case of PSI-LHCI super complex, the strategy evolved was to target photo-oxidation damage to the LHCI moiety to save the reaction center. This degradation also causes a decrease in PSI antenna size thus reducing light absorption efficiency and thus further decreasing the probability of damages. When the whole photosynthetic electron transport chain is considered, the strong resistance of PSI catalytic activity also appears well suited to prevent over-reduction of the plastoquinone pool and consequent PSII saturation and photoinhibition.

In line with the proposed role of LHCI as safety valve, in two previous studies the functional antenna size in PSI was evaluated *in vivo *after photoinhibitory treatment affecting PSI. Both studies show that a higher light intensity was required to oxidize P700, suggesting a reduction of the antenna proteins associated to PSI [42,50].

### The role of "red absorption forms"

This ability of LHCI to work as a safety valve for PSI is likely due to the presence in this antenna of the red absorption forms. These Chls are peculiar since they have energy level lower with respect to all other pigments in Photosystem I, including the reaction centre P700. Even at room temperature, 90% of the energy is concentrated in these chlorophylls before being transferred to PSI reaction centre [51]. This energy distribution explains why light in excess is affecting the antenna moiety rather than the reaction center: the probability of generating Chl triplets by intersystem crossing is clearly higher here than anywhere else in the complex.

**Figure 5:**
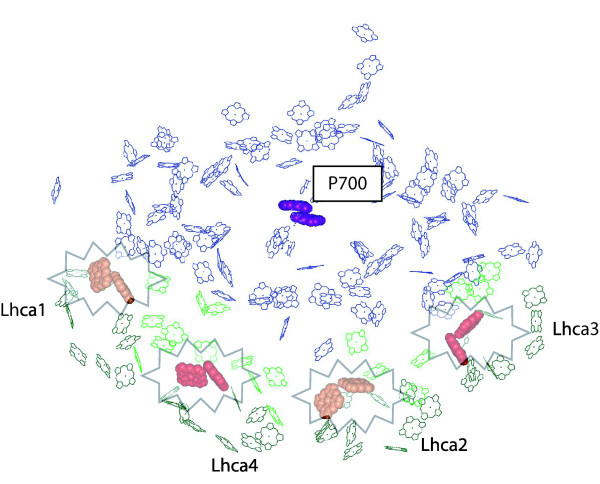
**The role of red forms in excitation energy localization**. Chl molecules of the structure from [41], deposited in the Protein Data Bank under accession number 1qzv, are shown. Core complex, antenna and linker chlorophylls are shown respectively in blue, dark and light green. The special pair P700 is highlighted in purple. Chls in sites 603 and 609 of antenna polypeptides are evidenced as well: they are in red in the case of Lhca3 and Lhca4, in orange for Lhca1 and Lhca2, according to their fluorescence emission properties [52].

In order to better understand the role of red Chls in the localization of excitation energy in excess far from the P700 it is useful to inspect the 3D structure of the complex [41]. In fact, red Chls have been identified to be Chls 603–609 (A5 - B5) in all Lhca1-4 complexes. Lhca3 and Lhca4 have the red-most absorption forms, while Lhca1-2 have relatively higher energies [52]. In figure 5, these chlorophylls are evidenced in PSI-LHCI structure: since energy is concentrated in these Chls and consequently most triplets are generated here, there is a very low probability of damaging the relatively far reaction center.

Red absorption forms, moreover, have some properties which make them particularly well photoprotected from excess light. In fact they have lower fluorescence yield with respect to all other antenna bound Chls, meaning they are particularly efficient in thermal dissipation of excess energy [53]. Furthermore, Chl triplets eventually generated by red Chls are efficiently quenched by nearby carotenoids [32]. This efficiency in Chl triplets quenching was also shown to be directly dependent on the presence of red forms and to be less efficient in a mutant depleted in red forms [32]. The presence of red Chls in PSI antenna is thus relevant for both photoprotective mechanisms evidenced in this work since (i) they contribute to increase the resistance of antenna proteins to high light and (ii) they localize radiation damages far from the reaction center.

### Differences and similarities in photoprotection strategies between PSI and PSII

Photosystem II is known to experience radiation damages even at very low illumination intensities. This is due to the fact that ^1^O_2 _is generated by charge recombination from P680^+ ^Pheo^- ^and that effective protection from carotenoids is lacking. In fact, carotenoid molecules in PSII core structure are located too far from P680 to be able to efficiently quench the Chl triplets generate by charge recombination [54]. On the other hand, if the carotenoids were closer, they would be immediately oxidized by P680^+ ^because of its strong oxidizing potential [54]. For this reason PSII reaction center is poorly protected from the action of singlet oxygen and an efficient mechanism of PSII repair has evolved in order to maintain the photochemical capacity. When light is in excess, the D1 protein is rapidly degraded and re-synthesized to reestablish a fully active PSII reaction center [6,55,56]. A similar mechanism is not known for PSI: after radiation damages its recovery requires several days [42,49,50]. Furthermore, the repair from photoinhibition does not require the turnover of a single polypeptide, as in the case of D1, but involves degradation and re-synthesis of the whole PSI complex.

Although the mechanisms and localization are different, the D1 protein might also be considered as a safety fuse: in fact, once D1 is degraded, not only charge separation but also singlet oxygen production are stopped thus protecting the remaining PSII core structures from oxidation damage and photobleaching [57]. Thus, it appears that in both cases (PSI and PSII) some sacrificial proteins exist functioning like a safety fuse for the rest of the structure. In the case of PSI these are the antenna chlorophyll-proteins while in the case of PSII is the D1 protein of the reaction center to play this role.

It is worth mentioning that P700^+ ^is not such a strong oxidizer as P680^+^, and no massive charge recombination has been reported in PSI. This is probably one major reason explaining why different photoprotection strategies evolved in PSI and PSII. It is interesting to point out that, despite the differences, in both cases the antenna system is fundamental in photoprotection. In PSII antenna function needs to be regulated: in low light conditions light harvesting capacity must be preponderant while in high light quenching mechanisms are activated [9]. Without such a regulation, PSII reaction center would be less efficient if associated to a constitutively quenched antenna, as suggested by the observation that plants over-expressing PsbS and accumulating zeaxanthin showed a reduced growth in limiting light conditions [12]. On the other side, PSI can afford being associated with an antenna with constitutive low fluorescence yield without losing efficiency in low light conditions [30].

The stability of PSI catalytic activity under high light also explains partially why PSII is widely considered as the only target of photoinhibition. In fact, early work in this field (reviewed in [5]) mainly evaluated the reaction center capacity of charge separation: for PSII this is easily affected by light treatments, while for PSI this occurs only under extreme conditions, as discussed above. A further possible explanation for the underestimation of LHCI role in photoprotection is also the fact that photoprotection capacity is not strongly modulated according to environmental conditions but rather intrinsic to the system. In fact, at present no modulation of light harvesting or fluorescence quenching efficiency was ever shown within the PSI antenna. Even the number of Lhca antennas associated with PSI is not reduced in high light conditions: four polypeptides, one copy of each Lhca1, 2, 3 and 4 polypeptide was in fact found associated to PSI in plants acclimated to different growing conditions, different from what was observed in the case of PSII antenna polypeptides [13].

## Conclusion

In this work we showed that the presence of the antenna system is effective in PSI photoprotection: if treated with strong illumination, PSI with reduced antenna showed a lower resistance than PSI-LHCI, both *in vivo *and *in vitro*. Antenna polypeptides not only increase the resistance of the supercomplex to light damages but also act as safety valves. Thanks to the presence of red forms, energy is concentrated far from the reaction centre, where any possible radiation damage will not affect catalytic activity.

## Methods

### Plant material and growth conditions

Wild type *Arabidopsis thaliana*, *lhca1 *(KO line, kindly provided by Prof. S. Jansson, Umea Sweden), *lhca3 *(antisense line NASC ID: N3892) and *lhca4 *mutants (T-DNA knockout line SALK-127744) are in the Col0 background, as described by [34,36]. *lhca2 *mutant (JIC Gene Trap line NASC ID: N101690; [58] was isolated in a Lansdberg erecta (Ler) background (see Additional file 1). For growth under controlled conditions six seeds from different *Arabidopsis thaliana *genotypes were sown in small pots and then stratified for 48 h at 4°C in the dark: Seeds were then transferred for 6–8 weeks in a growth chamber (22°C day/18°C night and 60% relative humidity) under a light cycle of 16 h of light (100 μE m^-2 ^s^-1^) and 8 h of dark.

### Lipid peroxidation Assay

The thiobarbituric acid (TBA) test, which determines malondialdehyde (MDA) as an end product, was used to analyze lipid peroxidation [37,59]. Briefly, detached leaves were treated with strong light (1000 μmol m^-2^s^-1^) at 4°C for 9 hours. Samples were harvested and then immediately frozen in liquid nitrogen. Photo-oxidative stress was assessed by measuring MDA formation, as indirect quantification of lipid peroxidation. MDA was quantified as MDA-(TBA)_2 _by HPLC as described [37].

### Purification of the native complexes

Unstacked thylakoid membranes were isolated from *Arabidopsis thaliana *leaves and used for PSI-LHCI particles purification. PSI-LHCI complex and its PSI core and LHCI moieties were then purified as previously reported [35,51].

### Photobleaching Assays

Kinetics of PSI-LHCI, PSI and LHCI photobleaching were measured by treating samples with 1600 μmol m^-2^s^-1 ^of white light (provided by a Schott KL1500 LCD) in a 1 cm cuvette water cooled at 10°C. Initial and maximal OD (at 435 nm) was 0.8 for wild-type PSI-LHCI particles, while average OD in whole visible spectra is 0.25. For figure 2 and 3, PSI complexes isolated from Lhca depleted plants, PSI core and LHCI, concentration was adjusted to an equivalent number of PSI reaction centers, by using the previously determined mutants antenna sizes [34,35]. Maximum OD employed are respectively 0.54 and 0.23 for isolated PSI core and LHCI. Experiments were also performed with equivalent Chl concentration (OD_435 _0.8 for all samples). A different photobleaching set-up was instead employed for gel electrophoresis and P700 determinations, since these analyses required larger sample amounts. PSI-LHCI samples (max OD_435 _2 per cm) in a 96 well plate were treated with 1000 μE m^-2^s^-1 ^from a white halogen lamp. The microtiter plate was kept in melting ice in order to control the temperature at 4°C. Optical length in the microtiter was 0.5 cm. For each point of the time course, samples from three different wells of the plate were pooled together and used for gel electrophoresis, pigment analyses and P700 measurements.

### SDS PAGE Electrophoresis

SDS-PAGE electrophoresis was performed as [60] with the modifications described in [46]: a acrylamide/bis-acrylamide ratio of 75:1 and a total concentration of acrylamide + bis-acrylamide of 4.5% and 15.5% were employed respectively for the stacking and running gel. 6 M urea was also incorporated into the running gel.

### Spectroscopy and pigment analysis

HPLC analysis was as in [61]. Chlorophyll to carotenoid ratio and Chl a/b ratio were independently measured by fitting the spectrum of acetone extracts with the spectra of individual purified pigments [62].

### Deriphat PAGE analysis

Non-denaturing Deriphat-PAGE was performed following the method developed by [63] with the following modifications: the stacking gel contained 3.5% (w/v) acrylamide (38:1 acrylamide/bis-acrylamide). The resolving gel was obtained with an acrylamide concentration gradient from 4.5 to 11.5% (w/v) stabilized by a glycerol gradient from 8% to 16%. 12 mM Tris and 48 mM Glycine pH 8.5 were also included in the gel. In the case of PSI-LHCI particles the equivalent of 10 μg of chlorophyll were loaded in each lane.

### Measurement of P700 redox kinetics

Functional P700 concentration in samples during the photobleaching experiments was estimated by measuring the light induced P700^+ ^absorption at 435 nm [64] using a laser based Joliot type spectrophotometer [33]. The measuring light pulse of 5 ns time-duration is produced by a Surelite Q-switched Nd:YAG laser (Continuum) which pumps a Surelite OPO (BBO I crystal, Continuum) to produce coherent, broadband tunable radiation from 420 to 590 nm. Excitation light is provided by dye laser emission (DCM 650) in a home built dye cell which is pumped by a second harmonic Minilite Nd:YAG laser (Continuum). The excitation pulses have an intensity of ~1.5 mJ, wavelength 640 ± 10 nm and duration of 5 ns. Three Schott BG39 (3 mm) optical filters and a low pass dielectric filter (transmission less than 10^-3 ^at 600 nm) are placed in front of each detector. Samples were placed in 2 mm cuvettes in front of each detector. The measurements were performed at room temperature. 1 mM ascorbate and 20 μM 5-methyl phenazonium methyl sulfate (PMS), were added to each sample just before measuring to assure P700 reduction in the dark. Data were fitted from 30 light induced absorption changes obtained in the time range from 1 ms to 1425 ms by a single decaying exponential function, the so obtained amplitude was taken as relative quantification of photo-oxidizable P700.

## Abbreviations

The abbreviations used are: α(β)-DM: *n*-dodecyl-α(β)-D-maltoside; Car: Carotenoid; Chl: chlorophyll; Lhca (b): light harvesting complex of Photosystem I (II); LHCI (II): antenna complex of Photosystem I(II); MDA: malondialdehyde; PSI (II): Photosystem I (II); ROS: Reactive Oxygen Species; WT: wild type.

## Authors' contributions

AA performed MDA as well as most photobleaching SDS-PAGE and non denaturing gel experiments; MB contributed to photobleaching, SDS-PAGE and non denaturing gel experiments; RH performed P700 kinetics experiments; GMG contributed to data analysis and writing; TM conceived experiments, analyzed data and wrote the paper. All authors read and approved the final manuscript.

## Supplementary Material

Additional file 1 **Characterization of the newly isolated Lhca2 KO plants**. a) Genomic DNA structures of Lhca2 alleles. Exons and introns are represented by boxes and lines, respectively. In lhca2.1, the Lhca2 gene (At3g61470) is disrupted by the insertion of the JIC T-DNA in between the second and the third intron. Below, an example of the segregation analysis using a specific antibody directed against Lhca2 polypeptide. A three week old plant was grinded directly in 50 μL loading buffer for total protein extraction and 5 μL of the extract used for western blotting analyses. *samples corresponding to lhca2 knock-out plants. b) Non denaturing gel electrophoresis of pigment binding complexes isolated from thylakoids of wild-type and Lhca depleted plants, after solubilization with final 0.8% α-DM. The equivalent of 25 μg of Chls was loaded for each sample. PSI-LHCI complex from this knew knock-out line has the same impact on complex stability as antisense line described in [35]. c) Sucrose density gradient profile of PSI and PSII super complexes. Super complexes of PSI-LHCI after thylakoid membrane solubilization with 1% β-DM. Complexes were collected from the gradient for spectroscopic characterization. In the black box the PSI-LCHI bands are highlighted. d) Absorption spectra of solubilized PSI-LHCI isolated from wild-type confirmed that the supercomplexes isolated from knock-out lines in Ler background are equivalent to previously characterized antisense lines in Col background. Click here for file

Additional file 2 **Chlorophyll leaf content in WT and plants depleted in different Lhca proteins**. Chl leaf content (ng per mg of fresh weight) is reported for all plants considered in this work. Data are the result of the measurement of at least 6 six week old leaves. Click here for file

Additional file 3 **Photobleaching experiments comparing PSI-LHCI and PSI core particles with equal chlorophyll concentration**. Photobleaching experiments were performed as in figure 2, comparing light sensitivity of PSI-LHCI from WT (black squares) with isolated PSI core (red circles), using samples with the same Chl concentration. Click here for file
