# Supplementary figures and images for: Antenna complexes protect Photosystem I from Photoinhibition
Source: BMC Plant Biol. 2009 Jun 9;9:71. doi: 10.1186/1471-2229-9-71 (PMC2704212; doi:10.1186/1471-2229-9-71)

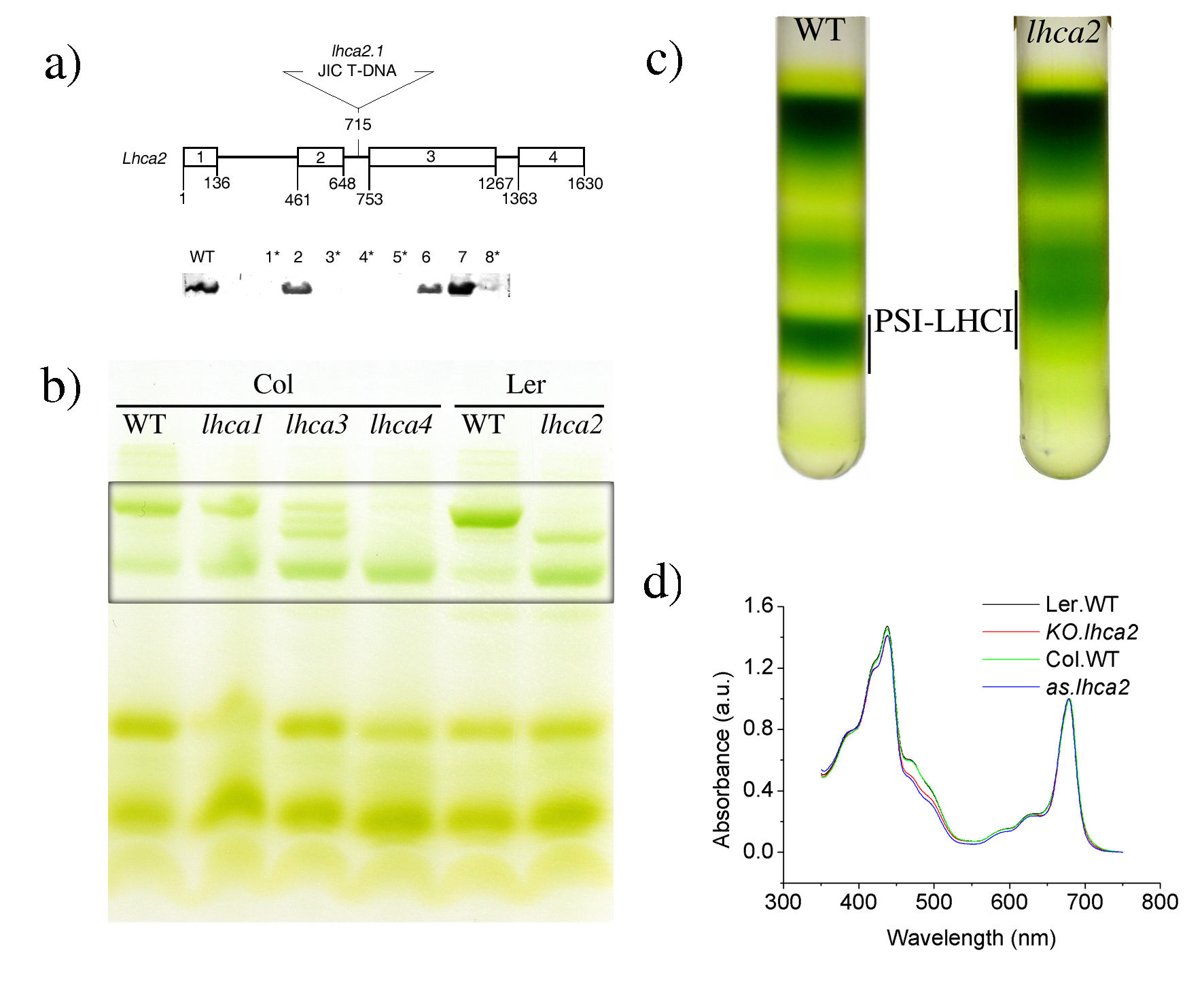

Supplement: Additional file 1 — Characterization of the newly isolated Lhca2 KO plants. a) Genomic DNA structures of Lhca2 alleles. Exons and introns are represented by boxes and lines, respectively. In lhca2.1, the Lhca2 gene (At3g61470) is disrupted by the insertion of the JIC T-DNA in between the second and the third intron. Below, an example of the segregation analysis using a specific antibody directed against Lhca2 polypeptide. A three week old plant was grinded directly in 50 μL loading buffer for total protein extraction and 5 μL of the extract used for western blotting analyses. *samples corresponding to lhca2 knock-out plants. b) Non denaturing gel electrophoresis of pigment binding complexes isolated from thylakoids of wild-type and Lhca depleted plants, after solubilization with final 0.8% α-DM. The equivalent of 25 μg of Chls was loaded for each sample. PSI-LHCI complex from this knew knock-out line has the same impact on complex stability as antisense line described in [35]. c) Sucrose density gradient profile of PSI and PSII super complexes. Super complexes of PSI-LHCI after thylakoid membrane solubilization with 1% β-DM. Complexes were collected from the gradient for spectroscopic characterization. In the black box the PSI-LCHI bands are highlighted. d) Absorption spectra of solubilized PSI-LHCI isolated from wild-type confirmed that the supercomplexes isolated from knock-out lines in Ler background are equivalent to previously characterized antisense lines in Col background. [file 1471-2229-9-71-S1.jpeg]

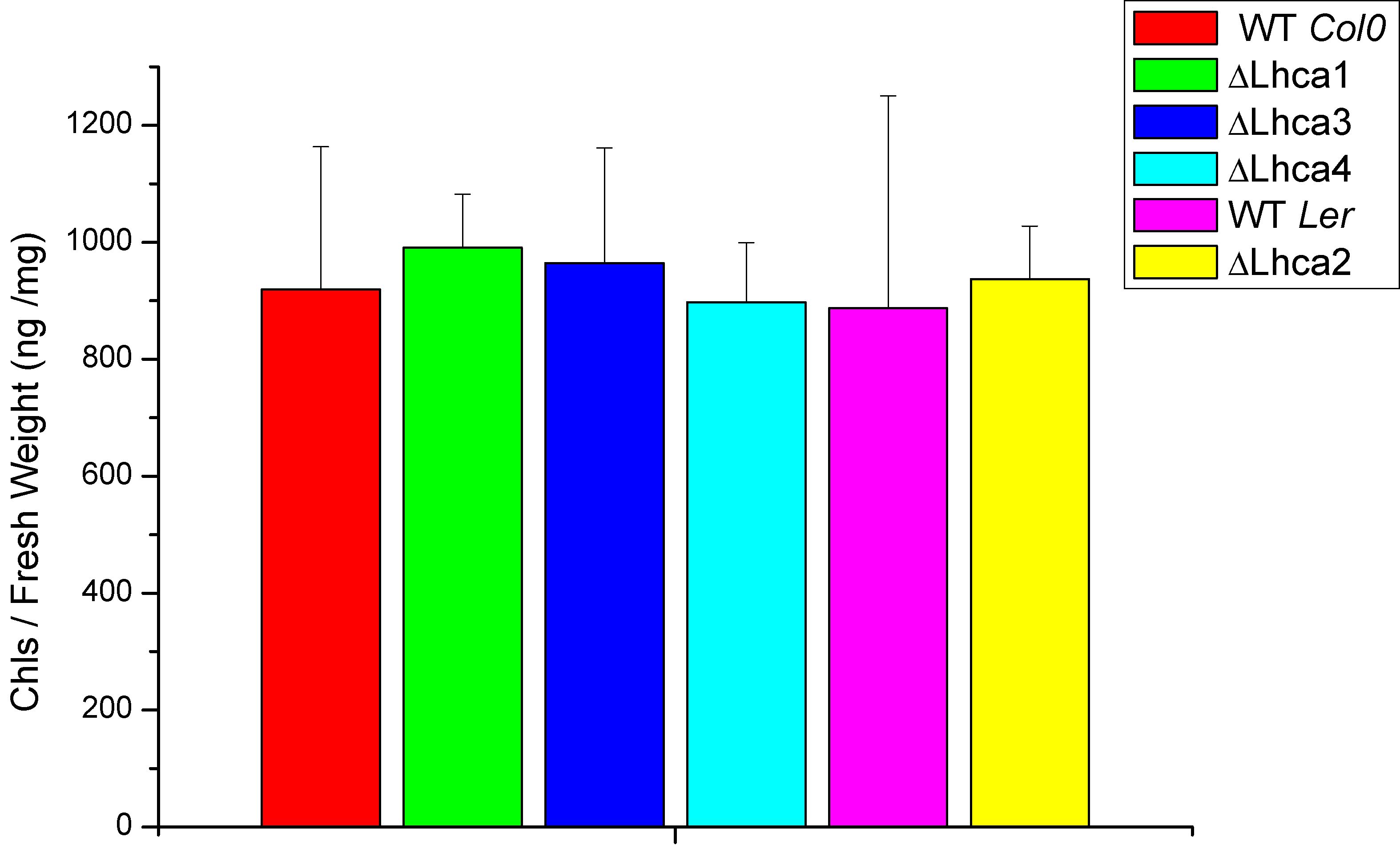

Supplement: Additional file 2 — Chlorophyll leaf content in WT and plants depleted in different Lhca proteins. Chl leaf content (ng per mg of fresh weight) is reported for all plants considered in this work. Data are the result of the measurement of at least 6 six week old leaves. [file 1471-2229-9-71-S2.jpeg]
